# Comparative Genomic Characterization of Three *Streptococcus parauberis* Strains in Fish Pathogen, as Assessed by Wide-Genome Analyses

**DOI:** 10.1371/journal.pone.0080395

**Published:** 2013-11-18

**Authors:** Seong-Won Nho, Jun-ichi Hikima, Seong Bin Park, Ho Bin Jang, In Seok Cha, Motoshige Yasuike, Yoji Nakamura, Atsushi Fujiwara, Motohiko Sano, Kinya Kanai, Hidehiro Kondo, Ikuo Hirono, Haruko Takeyama, Takashi Aoki, Tae-Sung Jung

**Affiliations:** 1 College of Veterinary Medicine, Gyeongsang National University, Jinju, Gyeongnam, South Korea; 2 Department of Biochemistry and Applied Bioscience, Faculty of Agriculture, Univeristy of Miyazaki, Miyazaki, Japan; 3 National Research Institute of Fisheries Science, Fisheries Research Agency, Yokohama, Kanagawa, Japan; 4 Faculty of Fisheries, Nagasaki University, Nagasaki, Japan; 5 Laboratory of Genome Science, Tokyo University of Marine Science and Technology, Minato, Tokyo, Japan; 6 Department of Life Science and Medical Bioscience, Waseda University, Shinjuku, Tokyo, Japan; 7 Consolidated Research Institute for Advanced Science and Medical Care, Waseda University, Shinjuku-ku, Tokyo, Japan; University of Kansas Medical Center, United States of America

## Abstract

*Streptococcus parauberis*, which is the main causative agent of streptococcosis among olive flounder (*Paralichthys olivaceus*) in northeast Asia, can be distinctly divided into two groups (type I and type II) by an agglutination test. Here, the whole genome sequences of two Japanese strains (KRS-02083 and KRS-02109) were determined and compared with the previously determined genome of a Korean strain (KCTC 11537). The genomes of *S. parauberis* are intermediate in size and have lower GC contents than those of other streptococci. We annotated 2,236 and 2,048 genes in KRS-02083 and KRS-02109, respectively. Our results revealed that the three *S. parauberis* strains contain different genomic insertions and deletions. In particular, the genomes of Korean and Japanese strains encode different factors for sugar utilization; the former encodes the phosphotransferase system (PTS) for sorbose, whereas the latter encodes proteins for lactose hydrolysis, respectively. And the KRS-02109 strain, specifically, was the type II strain found to be able to resist phage infection through the clustered regularly interspaced short palindromic repeats (CRISPR)/Cas system and which might contribute valuably to serologically distribution. Thus, our genome-wide association study shows that polymorphisms can affect pathogen responses, providing insight into biological/biochemical pathways and phylogenetic diversity.

## Introduction

Streptococcosis of cultured fish contributes to major economic losses in the aquaculture industries of Israel [1], Italy [2], Korea [3], Japan [4], and the United States [5]. *S. parauberis* was first reported from turbot (*Scophthalmus maximus*) cultured in Spain [6]; since the turn of the present century, it has become important disease in the aquaculture industries of Northeast Asia (Korea, Japan and China), especially among olive flounder aquaculture farms. Recently, Nho et al. [7] reported that *S. parauberis* is the dominant etiological agent of streptococcosis characterized by clinical symptoms such as chronic wasting syndrome, hemorrhagic septicemia, exophthalmia and meningitis with abnormal swimming.

*S. parauberis* exhibits serological, genetic and biochemical variations within the species. Nho et al [7] classified *S. parauberis* into three serotypes based on specific antigenic bands seen on Western blot analysis using anti-*S. parauberis* chicken IgY. In contrast, Kanai et al. [8] differentiated *S. parauberis* into two serotypes based on the chemical composition of a surface capsular polysaccharide. These variations may reflect an evolutionary trend, or may suggest that there is a relationship between serotype and virulence.

Comparative genome sequence analysis of bacteria can detect sequence diversity among distinct yet closely related populations, which may have important implications among the strains are important for adaptation, strain specific genes are thought to represent the physiological and virulence properties of an organism, disease epidemiology and understanding evolutionary relationships [9,10]. These genetic variations can arise from gene mutations, insertions, deletions and/or genetic noise. Such analyses have been performed for various pathogenic bacteria, including *Helicobacter pylori* [11], *Mycobacterium species* [12], *Escherichia coli* [13], *S. mutans* [14] and the fish pathogen *Edwardsiella tarda* [15]. Moreover the recent determination of the complete genomic sequence for *S. parauberis* revealed important information on bacterial diversity, functional characteristics, environmental adaptation, and virulence components [16].

Bacteria utilize diverse carbohydrate sources for various biosynthetic processes, particularly those involved in maintenance and reproduction. Many catabolic operons are subject to carbon catabolite repression (CCR) by rapidly metabolizable carbon sources, especially glucose [17]. In Gram-positive bacteria with low GC contents, however a very different CCR pattern is used for PTS sugar transport by EI-P on HPr residue histidine, and HPr can also be phosphorylated at serine 46 (Ser-P) by a specific ATP-dependent protein kinase [18]. Then, HPr (Ser-P) interacts with CcpA, which binds to a conserved sequence in the promoter region called the catabolite-responsive element, thereby negatively controlling the expression of several carbohydrate catabolism related enzymes [19,20]. 

Bacteria have evolved various mechanisms to defend themselves against viral predation. One such strategy involves the CRISPR/Cas systems, which are small RNA-based defense systems that provide adaptive, heritable immunity against viruses, plasmids and other mobile elements in archaea and bacteria [21]. The RNA and protein components of these immune systems arise from the CRISPR locus and the *cas* genes. The six *cas* genes (*cas*1-6) are present in a wide array of bacteria [21–24]. Most bacteria have some but not all of the *cas* genes; however, *cas*1 and *cas*2 appear to be universal and can therefore be used as genomic markers for the CRISPR/Cas system. 

In this context, we determined the whole genome sequences of two strains of *S. parauberis* (KRS-02083 and KRS-02109, representing serotypes I and II, respectively), that were isolated from Japan in 2002 and classified using a rabbit anti-serum based agglutination test [8]. We compared the genome sequences, genomic structures, and gene variations of these strains with those of a reference strain of serotype I (KCTC11537) isolated from South Korea in 2006. Our results show differentiation for carbon source utilization and phage resistance according geographical and serological strains which provide useful information on evolutionary events in the three *S. parauberis* strains and offer new insights into streptococcal species-specific survival and potential prophylactic strategies.

## Materials and Methods

### Bacterial strains

*S. parauberis* strains KRS-02083 and KRS-02109 were isolated from olive flounder at commercial aquaculture sites in Western Japan and grown in Todd Hewitt (TH; Oxoid Ltd., Cambridge, UK) broth (THB) or agar (THA) [8], at 25°C for 24 h. For long-term storage, bacteria were kept in THB containing 10% (v/v) glycerol at -70°C. The control, *S. parauberis* KCTC 11537 (GenBank accession no. NC_015558), was as previously described [16].

### Preparation of genomic DNA

The two *S. parauberis* strains were cultured in THB at 25°C for 24 h, and genomic DNA was extracted using the Qiagen Genomic-tip 500/G kit (Qiagen, Hilden, Germany) and a genomic DNA buffer set (Qiagen), according to the manufacturer’s instructions.

### Whole-genome sequencing

The draft genomes of *S. parauberis* KRS-02083 and KRS-02109 were sequenced by the National Research Institute of Fisheries Science (Yokohama, Japan) using a Roche/454 GS-FLX^TM^ system [25], and Takara Bio, Inc. (Otsu, Japan) using Illumina Genome Analyzer (GA) [26]. Large contigs were assembled using the Newbler *de novo* assembler package in the 454 GS-FLX^TM^ system, and pair-end reads were assembled with respect to the reference genome using BWA-SAMtools [27] and Edena [28] in Illumina GA. 

### Annotation and comparative multiple alignment analyses

The KRS-02083 and KRS-02109 genome sequences were annotated using the automated prokaryotic annotation server, Rapid Annotations using Subsystems Technology (RAST; rast.nmpdr.org) [29], and identified by manual NCBI BLAST searches. We evaluated the annotation accuracy by comparing the RAST results from our large scaffolds and pair-end reads to the coding sequences (CDSs) published reference genome (KCTC11537). Comparative circular maps of the genomes were constructed using the BRIG (BLAST Ring Image Generator) [30]. Homologies in the gene contents of the two genomes were identified using the comparative tools of the RAST algorithm, to allow for potential genome-to-genome annotation. CRISPR elements were identified and CRISPR-associated genes were annotated using CRISPRFinder [31] and our CDS results. Multiple alignments were performed using CLUSTALW [32], and various other comparisons and investigations were performed using the *in silico* molecular cloning tool, Genomic Edition Version 4.2.21 (In Silico Biology, Yokohama, Japan).

### PCR-based assay

The presence of each lactose-, sorbose- and glucose-associated gene was determined by PCR using specific primer sets (Table S1); the tested genes included *lac*C, *lac*E and *lac*G for lactose, *sor*C and *sor*E for sorbose, and GCK and *pts*G for glucose utilization. PCR detection of the *cas*1 gene was also performed for identification of CRISPR/Cas. The 24 stored *S. parauberis* strains utilized in this PCR-based assay had been isolated from diseased olive flounder in Korea (six strains each of type I and type II) between 2006 and 2009, and in Japan (six strains each of type I and type II) between 2002 and 2004. The isolates were cultured in TSB (for the Korean strains) and THB (for the Japanese strains) at 25°C for 24 h, and DNA was extracted using an AccuPrep® Genomic DNA Extraction Kit (Bioneer, Korea) according to the manufacturer’s instructions. PCR was performed in 20 μL reaction mixtures containing 1 μL template DNA, 0.05 μM of each primer (Bioneer), and the AccuPower PCR^®^ premix (Bioneer). Amplification was performed using a C-1000TM thermo cycler (Bio-Rad). The PCR conditions included an initial denaturation cycle of 94°C for 3 minutes; 30 cycles of denaturation step at 94°C for 30 seconds, annealing at 54°C for 30 seconds, and extension at 72°C for 30 seconds; and a final extension step at 72°C for 5 minutes. The resulting PCR products were analyzed on a 1.5% (w/v) agarose gel and stained 1% (w/v) ethidium bromide. DNA bands were visualized using a Gel Dock system (ATTO E-Graph, AE-9000; Takara, Japan) equipped with a CS analyzer program.

### Analysis of growth curves and sugar utilization

KRS-02083, KRS-02109 and KCTC11537 were maintained as previously described [8,16]. Bacterial cells were cultivated in TYE broth (tryptone, 10 g; yeast extract, 5 g; and K2HPO4, 2 g per liter) [33] supplemented with 20 mM glucose and 25 mM lactose and L-sorbose for the two Japanese strains and the Korean strain. The growth phenotypes of various strains were monitored using an xMark Microplate Absorbance Spectrophotometer (Bio-Rad, Hercules, CA, USA) at 37°C, with the optical density at 600 nm recorded every hour.

### Nucleotide sequence accession numbers

The whole genome sequences of KRS-02083 and KRS-02109 have been deposited in the GenBank database under accession numbers ALYM00000000 and ALWR00000000, respectively.

## Results and Discussion

### General genomic features

**Figure 1:**
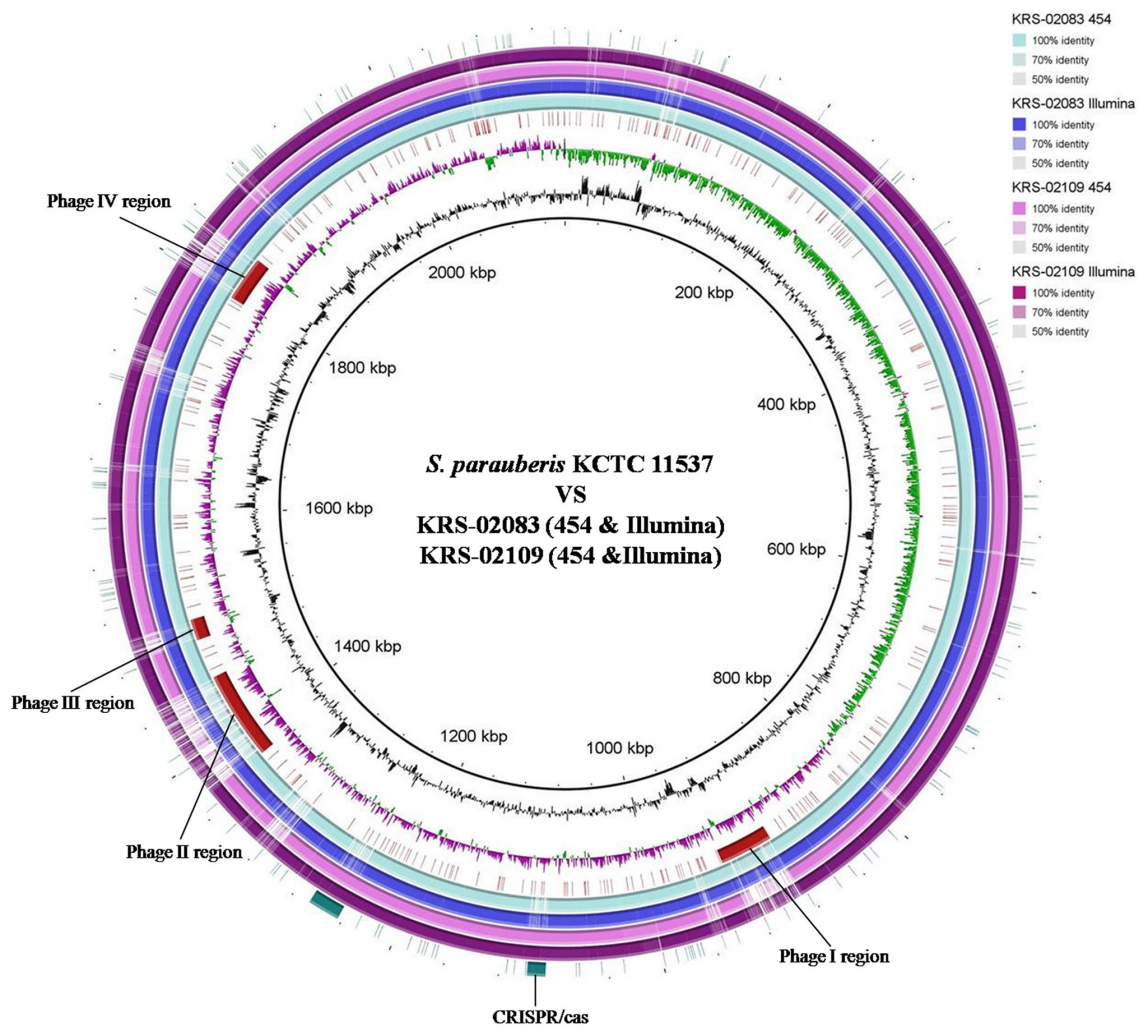
Circular maps from NGS comparing the genomes of *S. parauberis* strains KRS-02083 and KRS-02109 with that of reference strain KCTC11537. Beginning with the outermost ring, ring 1 shows the CRISPR/Cas and Tn916 regions encoded in the KRS-20083 genome. Rings 2 and 3 show large contigs from 454 GS-FLX and pair-end reads from the Illumina GA alignment of KRS-02083. Rings 4 and 5 show large contigs from 454 GS-FLX and pair-end reads from Illumina GA alignment in KRS-02109. Ring 6 shows four phage-associated regions in the KRS-02083 genome. Rings 7 and 8 show the GC content and GC skew, respectively, with respect to reference strain KCTC11537. The inner circle shows the scale (bp).

*De novo* assemblies of the KRS-02083 and KRS-02109 genomes were generated using two next generation sequencing technologies: the Illumina GA and GS-FLX pyrosequencing. The BRIG program [30] was used to compare the genomes of the *S. parauberis* strains via genomic alignment (Figure 1). 

The 650 ~750 bp and 580 ~660 bp reads were found to be the dominant lengths in the GS-FLX results for KRS-02083 and KRS-02109, respectively. The Illumina GA results comprised more than 10,000 contigs, including numerous small contigs (≤ 500 bp) and various contigs of different sizes (≥ 500 bp, ≥ 1kb, ≥ 2kb and ≥ 4kb) in both KRS-02083 and KRS-02109. The assembled sequence data for KRS-02083 and KRS-02109 yield coverages of 65.5 and 71.6 times that of the paired-end mapped reads from GS-FLX and, 500 and 600 times that of the single reads produced using the Illunina GA, respectively. In terms of the raw data, the KRS-02083 and KRS-02109 genome sequences consisted of 55 and 31 contigs, respectively, generated by GS-FLX sequencing, and 265 and 192 contigs, respectively, generated using Illumina GA. The KRS-02083 genome was estimated to be 2,126,607 bp in length with 88.61% nucleotide similarity to the reference genome and a GC content of 35.6%, while the KRS-02109 was estimated to be 2,084,517 bp in length with 87.35% nucleotide similarity to the reference genome and a GC content of 35.5%. 2,236; 2,048 CDSs were identified from the KRS-02083 and KRS-02109 genomes, respectively, by RAST annotation. The KRS-02083 and KRS-02109 genome sequences had 13 and 6 gaps, respectively, ranging in lengths from 500 bp to 40 kb (Table 1).

**Table 1:** Overall features of the *S. parauberis* KRS-02083 and KRS-02109 genomes compared with that of strain KCTC11537.

**Parameter**		**KCTC11537 (ref)**	**KRS-02083**	**KRS-02109**
**Illumina GA**	**Total no. of bases (b)**	-	1,043,833,490	1,290,529,310
	**No. of assembled reads**	-	29,823,814	36,872,266
	**Length of assembled reads (b)**	-	71	71
	**No. of total contigs**	-	265	192
	**No. of unmapped contigs**	-	138	63
**454 GS-FLX**	**Total no. of bases (b)**	75,170,197	130,986,679	143,264,028
	**Length average (b)**	523	586.97	563.39
	**No. of total contigs**	181	55	31
**RAST annotation (CDSs)**		2204	2,236	2,048
**RAST annotation (RNAs)**		69	68	78
**No. of gaps**		0	13	6
**GC contents (%)**		35.6	35.55	35.48
**Length (b)**		2,143,887	2,126,607	2,084,517

Approximately 70.7% and 72.3% of the *S. parauberis* KRS-02083 and KRS-02109 proteins, respectively, were grouped into 27 functional groups, and the remaining 29.3% and 27.7%, respectively, were assigned to the “unknown function” group, which contained the highest proportion of annotated genes. An overview of the functional annotation in the two *S. pararuberis* strains is shown in Table 2. The category of carbohydrate related proteins contain the highest percentage of known genes from the RAST database, at 14.8% and 15.6% for KRS-02083 and KRS-02109, respectively. These proteins are important to the ability of the bacteria to survive in their host’s complex environmental niche. The annotated genes of KRS-02083 and KRS-02109 also included genes involved in RNA metabolism (3.6% and 3.8%), protein metabolism (6.7% and 6.3%), DNA metabolism (5.2% and 4.8%) for carbohydrate metabolism and processing. These findings may reflect that the organism is well adapted to aquatic ecosystems containing a wealth of carbohydrates and nucleic acid. We also observed relatively high levels of genes involved in regulation and cell signaling, perhaps reflecting the capacity of these strains to cope with various growth conditions and stresses [34]. In contrast, we did not identify genes involved in the photosynthesis, iron acquisition and metabolism, secondary metabolism or nitrogen metabolism.

**Table 2:** Functional annotation of CDSs found in the KRS-02083 and KRS-02109 genomes, according to the RAST annotated categories.

**Functional category**	**KRS-02083**	**KRS-02109**
Cofactors, vitamins, prosthetic group, pigments	101 (4.5%)	102 (5.0%)
Cell wall and capsule	123 (5.5%)	117 (5.7%)
Virulence, disease and defense	36 (1.6%)	37 (1.8%)
Potassium metabolism	3 (0.1%)	3 (0.1%)
Photosynthesis	0 (0%)	0 (0%)
Miscellaneous	82 (3.7%)	84 (4.1%)
Phages, prophages, transposable elements, plasmids	30 (1.3%)	0 (0%)
Membrane transport	39 (1.7%)	39 (1.9%)
Iron acquisition and metabolism	0 (0%)	0 (0%)
RNA metabolism	80 (3.6%)	77 (3.8%)
Nucleosides and nucleotides	87 (3.9%)	83 (4.1%)
Protein metabolism	150 (6.7%)	129 (6.3%)
Cell division and cell cycle	22 (1.0%)	22 (1.1%)
Motility and chemotaxis	2 (0.1%)	1 (0.0%)
Regulation and cell signaling	22 (1.0%)	21 (1.0%)
Secondary metabolism	0 (0%)	0 (0%)
DNA metabolism	117 (5.2%)	99 (4.8%)
Fatty acids, lipids and isoprenoids	75 (3.4%)	76 (3.7%)
Nitrogen metabolism	0 (0%)	0 (0%)
Dormancy and sporulation	1 (0.0%)	1 (0.0%)
Respiration	38 (1.7%)	37 (1.8%)
Stress response	67 (3.0%)	63 (3.1%)
Metabolism of aromatic compounds	3 (0.1%)	3 (0.1%)
Amino acids and derivatives	124 (5.5%)	123 (6.0%)
Sulfur metabolism	8 (0.4%)	5 (0.2%)
Phosphorous metabolism	39 (1.7%)	38 (1.9%)
**Carbohydrates**	**331 (14.8%)**	**320 (15.6%)**
Unknown function	656 (29.3%)	568 (27.7%)
Total	2236 (100%)	2048 (100%)

### Conformation of SNPs and INDEL genes

A major goal for sequencing is to identify large and small changes in the genome that could explain the divergence of a strain or have implications for gene function. Therefore, we conducted a detailed analysis of single nucleotide polymorphisms (SNPs), variations in repeat numbers, and gene-scale gains or losses. Analysis of Illumina GA data allowed us to identify 5,360 and 4,241 single mutations in the genomic sequence of KRS-02083 and KRS-02109, respectively, compared with the reference genome. 

**Figure 2:**
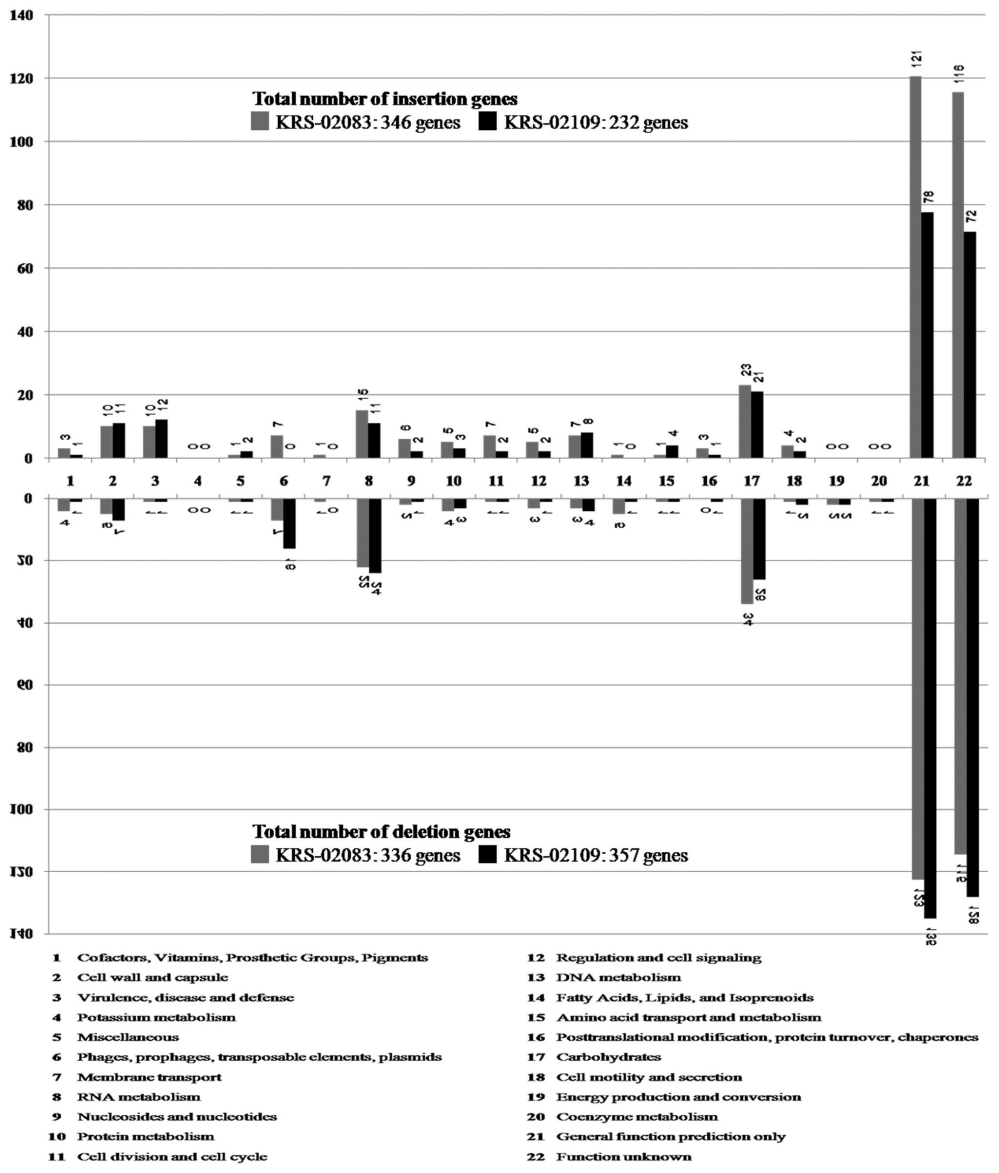
Comparison of gene contents and numbers with reference strain KCTC11537. As assessed by RAST functional categories grouped among insertion/deletion genes, between the KRS-02083 and KRS-02109 *S. parauberis* genomes.

Gene insertion-deletion (INDEL) events were abundant in the KRS-02083 and KRS-02109 genomes, which contained 346 and 232 were newly annotated genes and lacked 336 and 357 genes, respectively, with respect to the KCTC 11537 genome. Most of INDEL genes were hypothetical genes or genes of unknown function (Figure 2). INDEL genes contribute to species diversity and might encode supplementary biochemical pathways and functions that are not essential for bacterial growth but may confer selective advantages, such as adaptation to different niches and/or antimicrobial resistance. Large INDELs of gene regions can also create structural variations of the chromosome; these may be used as genotype-specific markers for epidemiological studies, and their study can offer a novel approach to understanding genetic diversity [35]. As such, our comparative analysis of the three *S. parauberis* genomes may provide important new insights into the evolutionary history of microbial pathogens and heterogenetic specificity in similar species.

### Lactose (lac) operon

**Figure 3:**
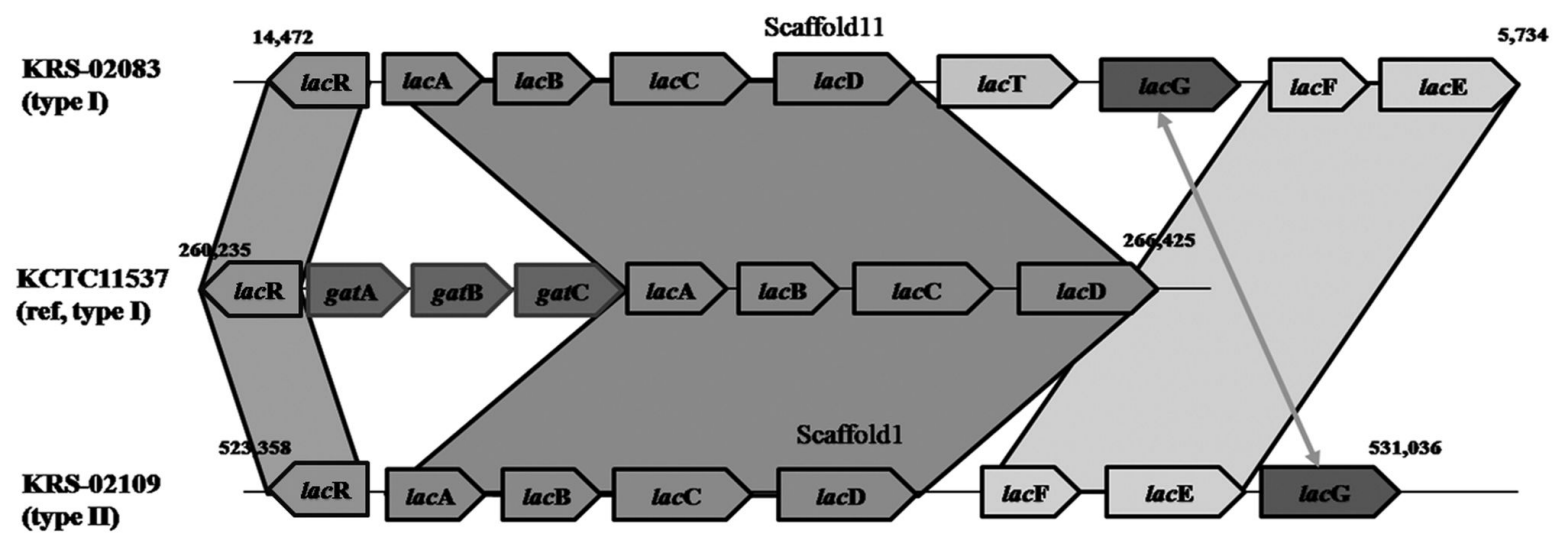
Structure variation of lactose operon among three *S. parauberis* strains. Comparison of *S. parauberis* KCTC11537, KRS-02083 and KRS-02109 reveals that lactose operons are sequence reservoirs for genetic diversity among strains. Schematic representation of the genetic characterization of lactose operons found in the three *S. parauberis* genomic sequences.

The KRS-02083 and KRS-02109 genomes contained *lac* operons homologous to those in *S. bovis* (NZ_AEEL01000000) and *S. pyogenes* (NC_002737.1) (Table 3). The KRS-02083 genome encoded *lac*RABCDTGF and E in scaffold 11 while KRS-02109 genome encoded *lac*RABCDFE, and G in scaffold 1 (Figure 3). The *lac*R determinant extended to the first ORF of the *lac* operon in both genomes which contained a DeoR-like helix-turn-helix domain and DeoR terminal sensor domain that detects diverse sugar derivatives in multiple genera, including tagatose phosphate in *S. mutans* [36]. In this regard, galactose-6-phosphate and tagatose-6-phosphate have been suggested to bind to the *lac*R cistron and facilitate its dissociation from the binding site, AGGAG. The adjacent genes in order, are galactose-6-phosphate isomerase A/B subunits, tagatose-6-phosphate kinsase and tagatose-1,6-diphosphate aldoase; designated as *lac*ABC and D. The *lac*A/B enzymes convert galactose-6-phosphate to tagatose-6-phosphate, has a broad specificity for various sugars, and can be used to produce rare sugars. Previously, Zeng et al. [36] showed that a mutant strain of *S. mutans* lacking *lac*A/B failed to grow on lactose supplemented medium, indicating that they are essential for the intracellular catabolism of lactose. However, further research is required to clarify the underlying mechanism. *Lac*C and *lac*D are preceded by a non-translated region containing the promoter along numerous direct and inverted repeats that are involved in regulating the *lac*-PTS operon. 

**Table 3:** List of lactose-associated genes encoded in the three *S. parauberis* genomes (KRS-02083, KRS-02109 and KCTC11537).

	**Gene function**	**KCTC11537**	**KRS-02083**	**KRS-02109**
	Lactose utilization (API20strep)	Negative	Positive	Positive
**1**	Lactose phosphotransferase system repressor (*lac*R)	STP_0241 (*S. equi* 73%)	SPJ1_2215 (*S. pyogenes* 99%)	SPJ2_0543 (*S. pyogenes* 99%)
**2**	PTS, galactose-specific IIA component (gatA)	STP_0242 (*S. uberis* 65%)	-	-
**3**	PTS, galactose-specific IIB component (*gat*B)	STP_0243 (*S. uberis* 91%)	-	-
**4**	PTS, galactose-specific IIC component (gatC)	STP_0244 (*S. uberis* 94%)	-	-
**5**	Galactose-6-phosphate isomerase A (*lac*A)	STP_0245 (*S. equi* 84%)	SPJ1_2214 (*S. pyogenes* 98%)	SPJ2_0544 (*S. pyogenes* 98%)
**6**	Galactose-6-phosphate isomerase B (*lac*B)	STP_0246 (*S. equi* 92%)	SPJ1_2213 (*S. pyogenes* 98%)	SPJ2_0545 (*S. pyogenes* 98%)
**7**	Tagatose-6-phosphate kinase (*lac*C)	STP_0247 (*S. gallolyticus* 65%)	SPJ1_2212 (*S. pyogenes* 95%)	SPJ2_0546 (*S. pyogenes* 95%)
**8**	Tagatose-1,6-bisphosphate aldolase (*lac*D)	STP_0248 (*S. gallolyticus* 84%)	SPJ1_2211 (*S. pyogenes* 96%)	SPJ2_0547 (*S. pyogenes* 96%)
**9**	Transcriptional antiterminator (*lac*T)	-	SPJ1_2210 (*S. bovis* 86%)	-
**10**	PTS, lactose-specific IIB component (*lac*E)	-	SPJ1_2207 (*S. bovis* 92%)	SPJ2_0549 (*S. pyogenes* 98%)
**11**	PTS, lactose-specific IIA component (*lac*F)	-	SPJ1_2208 (*S. bovis* 100%)	SPJ2_0548 (*S. pyogenes* 96%)
**12**	6-Phospho-beta-galactosidase (*lac*G)	-	SPJ1_2209 (*S. bovis* 99%)	SPJ2_0550 (*S. pyogenes* 99%)

Symbols: parentheses indicate the sequence homology in NCBI; ‘- ’ indicates non-coding.

The next sets of genes in the *lac*-PTS operons of the two tested strains were *lac*TGFE in KRS-02083, and *lac*FEG in KRS-02109, which are similar in structure to that in *Lactobacillus rhamnosus* [37], and *S. mutans* [36], respectively. The *lac*F and *lac*E genes were clearly related to the hydrophilic PTS IIA and PTS IIB components. *Lac*E in particular was found to have the predominantly hydrophobic N-terminal region typical of integral membrane proteins, followed by a hydrophilic domain that resembled the cytoplasmically oriented PTS lactose-specific IICB component [38]. The enzyme II components of the lactose PTS in the studied Japanese *S. parauberis* strains respond to intracellular inducers: IICB (*lac*E) and free cytosolic IIA (*lac*F), these domains may be part of a single polypeptide or may exist as separate interacting proteins [39]. The two Japanese *S. parauberis* genomes also contain 6-phospho-beta-galactosidase (*lac*G), an enzyme essential for the catabolism of lactose phosphate into glucose and galactose-6-phosphate, important for lactose hydrolysis (Figure S1). It has been suggested that in some cases, lactose 6-phosphate can be hydrolyzed by beta-glycosidases that are specific for beta-glucoside sugars. The absence of *lac*G in KCTC11537 explains the previous finding that it is unable to grow on lactose [36].

Thus, in the two Japanese *S. parauberis* genomes, the *lac*ABCD genes comprise the tagatose-6-phosphate pathway and are co-transcribed with genes *lac*FE and G, which specify proteins for the transport and cleavage of lactose. Our findings support the previous reports that KRS-02083 and KRS-02109 showed positive reactions to lactose acidification in biochemical tests, whereas KCTC11537 did not [7,8]. 

### Sorbose operon

**Figure 4:**
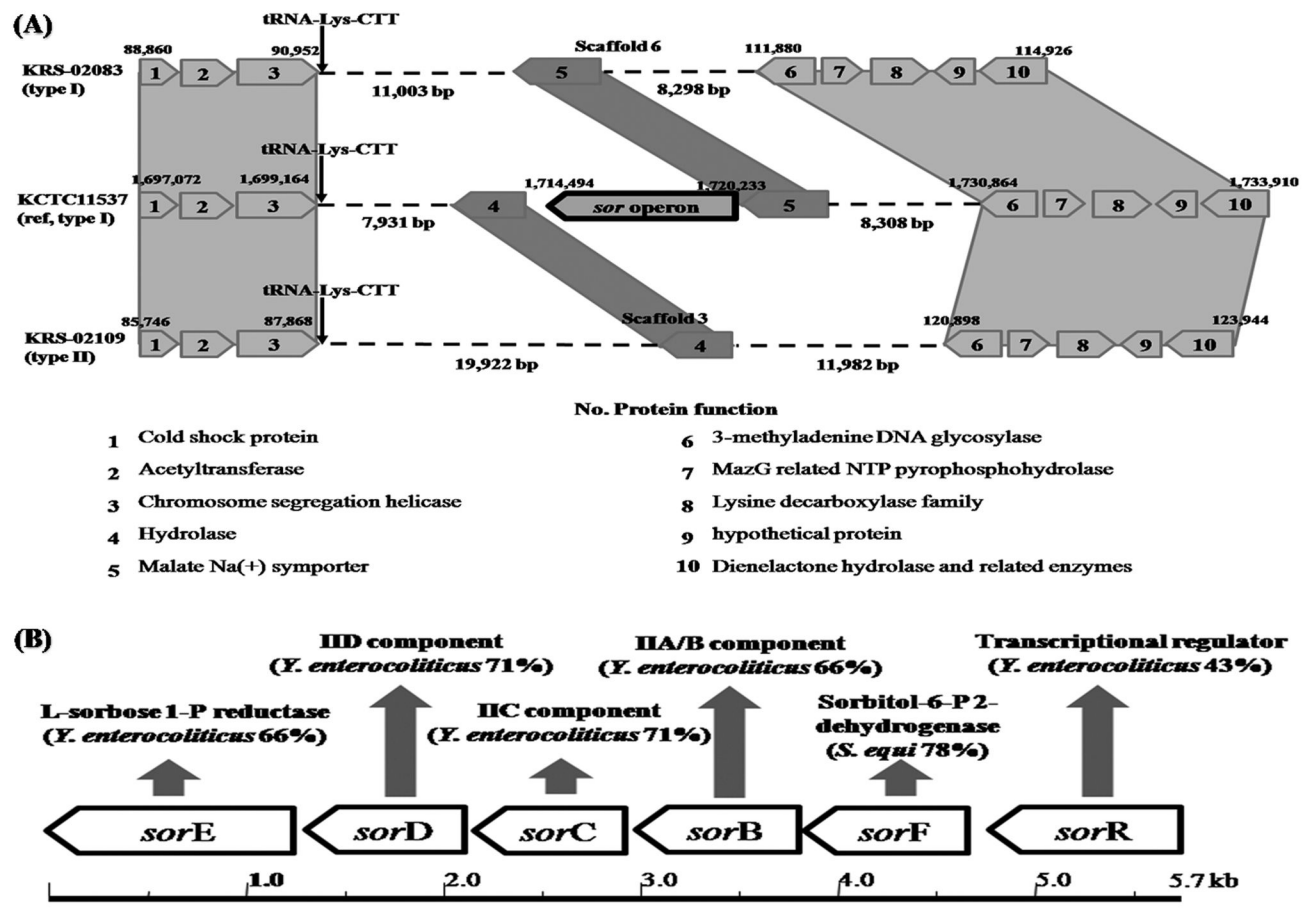
Structure variation of L-sorbose operon among three *S. parauberis* strains. (A) Comparison of *S. parauberis* KCTC11537, KRS-02083 and KRS-02109 reveals that sorbose operons are sequence reservoirs for genetic diversity among strains. (B) Simplified restriction map and genetic organization of the sorbose operon from *S. parauberis* KCTC11537.

Sorbose is a known carbon energy source in *E. coli* [40]. KCTC11537 showed a positive reaction to sorbose acidification test [7,8], and encodes L-sorbose utilization factors (*sor*EDCBF and R) with a 5.7-kb DNA fragment (Figure 4A). Intracellularly, L-sorbose-1-phosphate is reduced to D-sorbitol-6-phosphate by an L-sorbose-1-phosphate reductase (*sor*E). Then, in an NAD^+^-dependent step, sobitol-6-phosphate dehydrogenase (*sor*F) catalyzes the oxidation of D-sorbitol-6-phosphate to D-fructose-6-phosphate, which is then further catabolized by glycolytic enzymes (Figure S1). Sequence analysis revealed the presence of six *sor*-utilization ORFs, similar to the *sor* operon of *Yersinia enterocolitica* [41] except for the absence of *sor*F in the KCTC11537 (Figure 4B). The predicted gene products also showed homologies to various PTS proteins, including the IIB, IIC and IID components of the sorbose-specific PTS, and the catalytic enzyme L-sorbose 1-phosphate reductase from *Y. enterocolitica* (39%, 71%, 71% and 66%, respectively). The last gene, *sor*R, showed 43% homology with the corresponding gene of *Y. enterocolitica*. 

In KCTC11537, the L-sorbose PTS EII consists of two membrane-bound proteins, IIC (*sor*C) and IID (*sor*D), and two soluble components IIA/B (*sor*B); BLAST searches suggested that these are fused into a single polypeptide chain, as in *E. coli* [42]. When the predicted amino acid sequence of *sor*R was used in a BLAST search, we found that its closest homologs included a transcriptional regulator from *Y. enterocolitica* [43], a DeoR family transcriptional regulator from *Sebaldella termitidis* [44] and a *sor* operon regulator from *E. coli* [45]. These results suggest that the helix-turn-helix is a major structural motif capable of binding DNA; composed of two alpha helices joined by a short strand of amino acids, this motif is found in many proteins that regulate *sor* gene expression [46]. KCTC11537 encodes two sorbitol 6-phosphate 2-degydrogenase genes, *sor*F and *srl*D, which were found to be 78% and 85% similar to those in *S. equi* [35]. This suggests that cross-talk occurs between the *sor*F and *srl*D regulatory metabolic pathways and the redox state of the cell, and further indicates that the genes involved in L-sorbose and D-sorbitol catabolism interact through common effectors to reduce sorbitol-6-phosphate by converting it to fructose-6-phosphate. 

### PCR-based survey of carbon utilization genes

The utilization genes for glucose, GCK and *pts*G, were detected as 480- and 875- bp bands, respectively (Table 4). Our results revealed that the major pathways of carbohydrate uptake in 24 *S. parauberis* isolates all involved the PTS. The nucleotide sequences of the GCK and *pts*G genes in the three genomes examined herein were highly homologous (over 99%) further supporting this contention. Glucose is the preferred carbon source in many bacteria, and the presence of glucose often prevents the use of other, secondary carbon sources (e.g., sorbose, lactose and many others) [47]. The lactose-utilizing genes, *lac*C, *lac*E and *lac*G, were amplified from 10 of the 24 tested isolates, including the Korean strain, J27, and the Japanese strains, KRS-02083, KRS-02030, KRS-02067, KRS-02109, KRS-02068, KRS-02090, KRS-02087, KRS-02091 and KRS-02102. Eleven of the 24 isolates were found to have genes for sorbose utilization, such as *sor*C and *sor*E; these isolates included the Korean strains, KCTC11537, KCTC11538, J19, J20, J24, J25 and J21, and the Japanese strains, KRS-02032, KRS-04024, KRS-02067 and KRS-04037. We failed to detect any sorbose- or lactose- associated genes in Korean strains J22, J23, J28 and J30 (Table 4). The KCTC11537 genome was found to include a sorbose-utilization, locus that contained the genes for the PTS EII domains that are involved in the transport of extracellular sorbose and the phosphorylation of sorbitol. The KRS-02083 and KRS-02109 genomes, in contrast, both encoded *lac* operons, but showed different gene sequences. 

**Table 4:** PCR amplification.

	**Name**	**Year**	**Serotype**	**lacC**	**lacE (J1)**	**lacG (J1)**	**lacE (J2)**	**lacG (J2)**	**sorC**	**sorE**	**GCK**	**ptsG**	**cas1**
**Korean strains**	**KCTC11537 (ref)**	2006	I	-	-	-	-	-	+	+	+	+	-
	KCTC11538	2008	I	-	-	-	-	-	+	+	+	+	-
	J19	2009	I	-	-	-	-	-	+	+	+	+	-
	J20	2009	I	-	-	-	-	-	+	+	+	+	-
	J24	2009	I	-	-	-	-	-	+	+	+	+	-
	J25	2009	I	-	-	-	-	-	+	+	+	+	-
	J21	2009	II	-	-	-	-	-	+	+	+	+	+
	J22	2009	II	-	-	-	-	-	-	-	+	+	+
	J23	2009	II	-	-	-	-	-	-	-	+	+	+
	J27	2009	II	+	-	-	+	+	-	-	+	+	+
	J28	2009	II	-	-	-	-	-	-	-	+	+	+
	J30	2009	II	-	-	-	-	-	-	-	+	+	+
**Japanese strains**	**KRS-02083**	2002	I	+	+	+	-	-	-	-	+	+	-
	KRS-03032	2003	I	-	-	-	-	-	+	+	+	+	-
	KRS-02030	2002	I	+	+	+	-	-	-	-	+	+	-
	KRS-04024	2004	I	-	-	-	-	-	+	+	+	+	-
	KRS-02067	2002	I	+	+	+	-	-	+	+	+	+	-
	KRS-04037	2004	I	-	-	-	-	-	+	+	+	+	-
	**KRS-02109**	2002	II	+	-	-	+	+	-	-	+	+	+
	KRS-02068	2002	II	+	-	-	+	+	-	-	+	+	+
	KRS-02090	2002	II	+	-	-	+	+	-	-	+	+	+
	KRS-02087	2002	II	+	-	-	+	+	-	-	+	+	+
	KRS-02091	2002	II	+	-	-	+	+	-	-	+	+	+
	KRS-02102	2002	II	+	-	-	+	+	-	-	+	+	+

Lactose-, sorbose- and glucose-related genes, along with gene markers of the CRISPR/Cas system, were PCR amplified from 24 stored *S. parauberis* strains that had been isolated from diseased olive flounder in Korean (six strains each of type I and type II) between 2006 and 2009, and Japan (six strains each of type I and type II) between 2002 and 2004.

Symbols: ‘-’ indicates no amplification; ‘+ ’ indicates amplification.

### Sugar utilization

**Figure 5:**
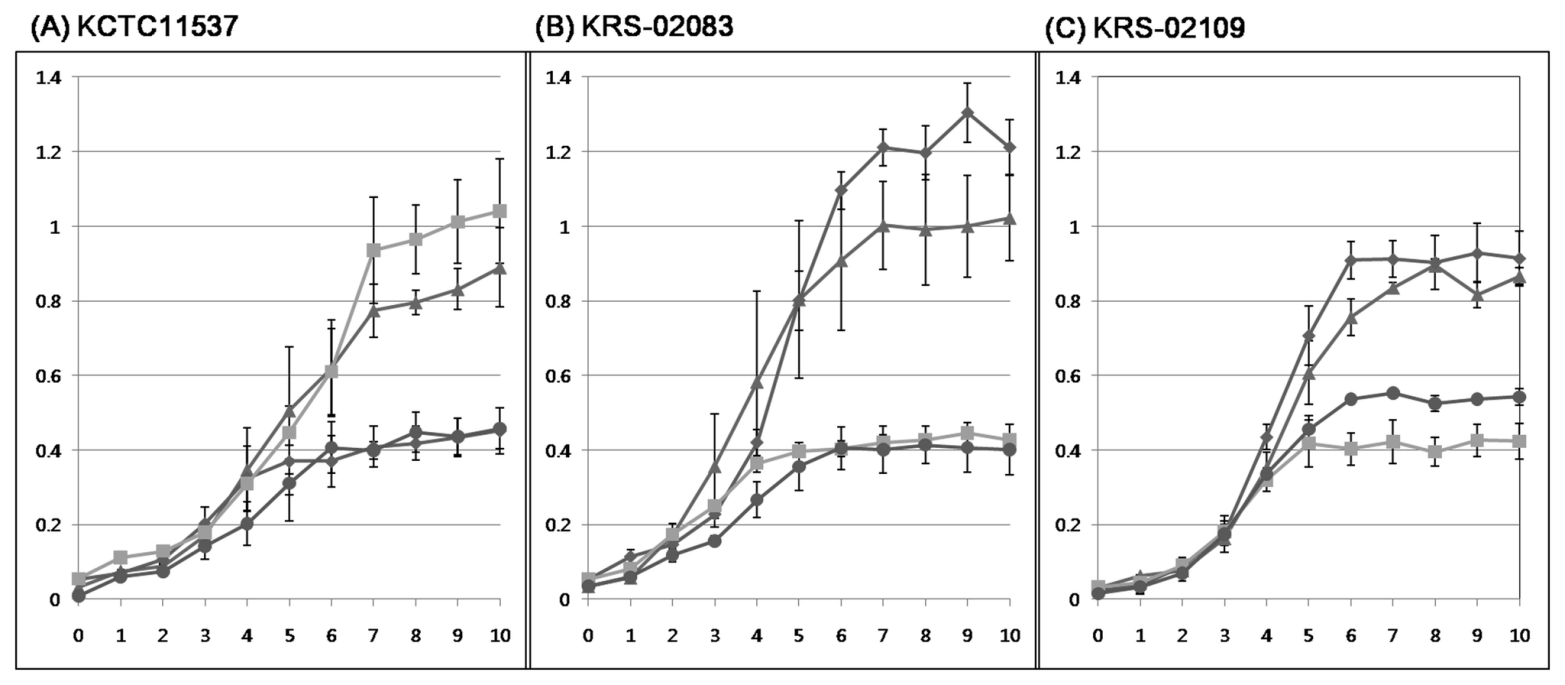
Growth curve abservation. Growth of KCTC11537 (A), KRS-02083 (B) and KRS-02109 (C) on TYE supplemented with glucose (▲), lactose (◆), sorbose (■), or a non-carbon source (●).

The difference in the encoded PTS components was confirmed when the bacterial strains were grown in TYE supplemented with D-glucose and with either lactose or L-sorbose. The two Japanese strains encoding the PTS components and tagatose associated enzymes involved in lactose metabolism grew in TYE supplemented with D-glucose and lactose. In contrast, the Korean strain encoding the PTS components and enzymes involved in sorbose metabolism grew well in TYE supplemented with D-glucose and L-sorbose; all strains show similar growth patterns on TYE broth containing non-carbon source (Figure 5). These results indicate that the PTS elements mediate the expression of the *lac* and *sor* operons within the cellular membrane. 

In many bacteria the amount of carbohydrates flowing through glycolysis is the primary trigger for binding of CcpA which is a CCR element that is crucial for sporulation [48,49], antibiotic resistance [50], and expression of virulence genes for pathogenicity [51]. It is important to keep in mind that the aim of pathogenic bacteria is to gain access to nutrients rather than to cause damage to the host. A number of papers have described that the carbon sources have effects on virulence particularly in terms capsule production to avoid phagocytosis and efficient attachment to host cells or tissues [52,53]. Differential nutrient availability within diverse host niches impacts upon the ability of *S. parauberis* cells to counteract local stresses and resist pharmacological intervention. To better understand how the catabolism of lactose and sorbose affect the virulence mechanisms in *S. parauberis*, future studies should examine the properties of various mutant strains lacking certain PTS-components, regulatory enzymes and other constituents of the catabolic pathways.

### CRISPR/Cas systems

**Figure 6:**
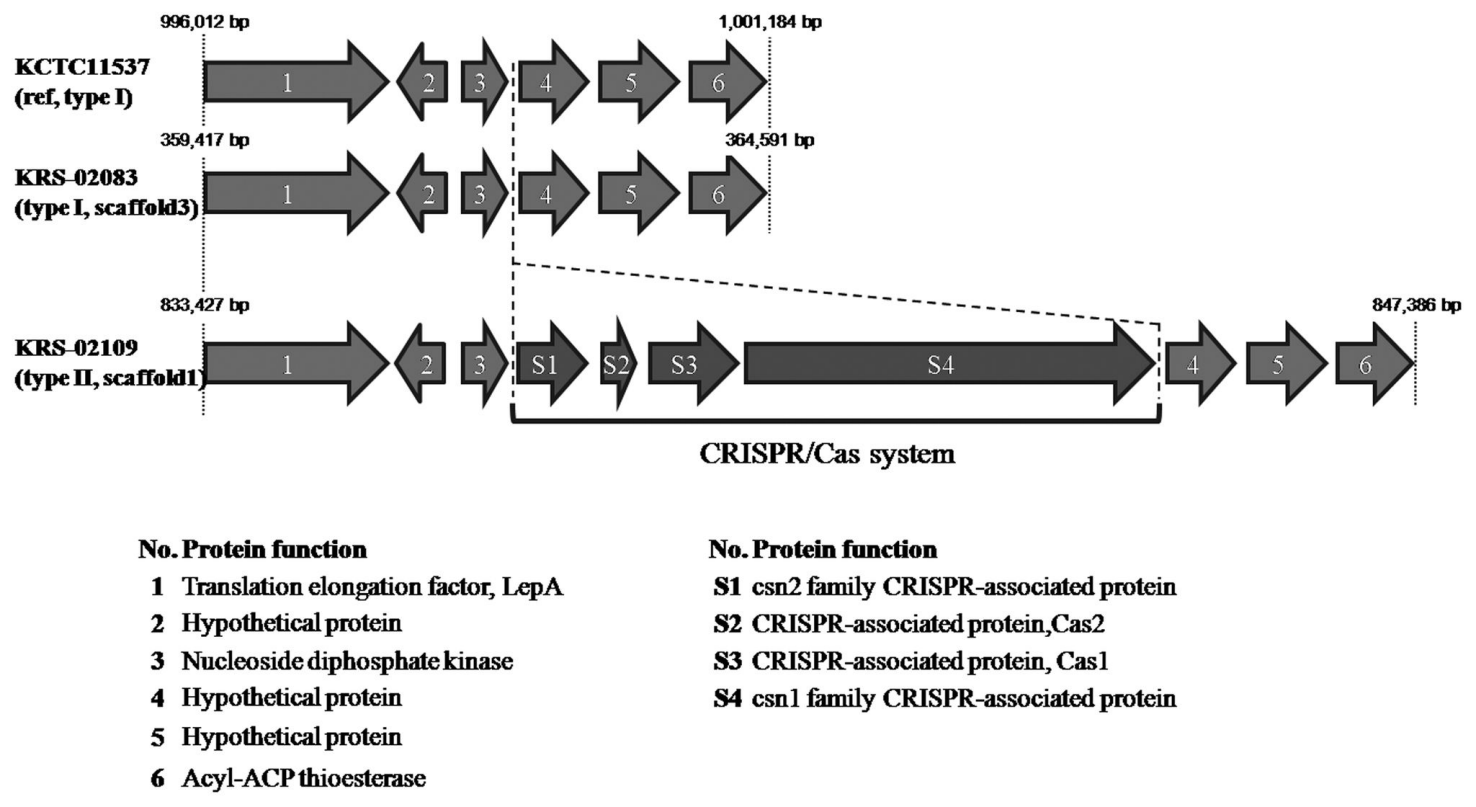
Genetic structure of the chromosomal region where the CRISPR/Cas system is integrated in KRS-02109.

In *S. pyogenes*, phage integration, as shown by genomic rearrangement of the prophage regions, is an important source for new virulence factors [54]. Mechanisms of phage resistance have not been previously described in *S. parauberis*, but genomic analyses have suggested that the CRISPR/Cas system helps provide adaptive immunity against foreign genetic elements in phages. Of the genomes examined herein, only KRS-02109 was found to possess characteristic CRISPR/Cas system; it contains genes highly similar to *cas*1, *cas*2, and two *csn* family genes *S. pseudoporcinus* (Figure 6). The *cas*1 gene encodes a metal-dependent DNA-specific endonuclease that may play a role in the recognition, cleavage, and/or integration of foreign nucleic acids into CRISPRs [55].

The biological significance of the CRISPR/Cas system against phage infection has been examined in *S. thermophilus* [54], where the presence of a CRISPR spacer identical to a phage sequence adjacent to *cas* provides resistance against phages containing that particular sequence (direct sequence consensus: GTT TTG GAA TCA TTC AAA ATA ACA TAG CTC TAA AAC). The spacer sequences found in the CRISPR loci of KRS-02109 showed sequence similarity to a lytic phage suggesting that *S. parauberis* KRS-02109 might resist phage infection via its CRISPR/Cas system. KCTC11537 and KRS-02083 each encode four phage associated loci in their genomes (Figure 1), suggesting that these four regions of the KCTC11537 and KRS-02083 genomes may be involved in the acquisition of foreign genes via natural transformation or bacteriophage activity. In contrast, no such phage-associated gene was found in the KRS-02109 genome, suggesting that this strain may resist phage infection through its CRISPR/Cas system. Interestingly, the *cas*1 gene was amplified from type II strains obtained from both Korea and Japan (Table 4), further suggesting that the CRISPR/Cas system might contribute to serology.

In the genus streptococcus, specific species may have evolved particular functions by acquiring foreign genes via natural transformation or bacteriophages. However, the acquisition of new foreign genes via phage infection may not be have always favored their lifestyles. In the future, elucidation of the mechanism by which *S. parauberis* acquires new genes could help clarify the species-specific evolutionary strategies among the streptococci. 

## Conclusion

Our genomic comparison of three *S. parauberis* strains revealed numerous biological, virulence, and pathogenetic factors, reflecting the organism’s adaptation as an obligate and versatile fish pathogen. The genomic features of the two Japanese strains (KRS-02083 and KRS-02109) were overall similar to those of the reference genome (KCTC11537). The *S. parauberis* genome appears to encode a number of carbohydrate related proteins (comprising the highest percentage of the annotated genes) and seems to confer the ability to synthesize all of the amino acids and regulatory factors required to survive in the host’s complex environmental niche. 

The genomes of the two Japanese strains each encoded a *la*c operon, which was found to be homologous in both type I and type II strains, and the loci were structurally similar to those of *L. rhamnosus* and *S. mutans*. In contrast, KCTC11537 exhibited a positive reaction to sorbose and was found to encode L-sorbose utilization factors. Its genome includes a 5.7-kb chromosomal DNA fragment that harbors six *sor* ORFs, which encode intracellular inducers and proteins capable of responding to external sorbose. Of the three examined strains, only KRS-02019 appears to preferentially use a CRISPR/Cas system to defend against phage integration. The *cas*1 gene, however, was amplified from all type II strains, regardless of geographic distribution.

Our genome sequence analyses showed that sequence diversities can exist among closely related, but distinct populations. We observed distinct variations, most notably in carbohydrate utilization, between similar species from different geographic regions, suggesting that the bacteria may have adapted to using certain carbohydrate sources that are abundant in particular area and/or utilizing secondary carbohydrate sources in the absence or lack of a primary source. Our findings may also be useful for the development of new prophylactic and therapeutic strategies to counter fish streptococcal infection.

## Supporting Information

Table S1 **PCR primers for this study.**
(DOCX)

Figure S1 **Sugar utilization.** Metabolic pathway in three *S. parauberis* for the conversion of lactose (two Japanese strains) and sorbose (A Korean strain) to glycosysis.
(DOCX)
