# Supplementary material for: Comparative Genomic Characterization of Three Streptococcus parauberis Strains in Fish Pathogen, as Assessed by Wide-Genome Analyses
Source: PLoS One. 2013 Nov 18;8(11):e80395. doi: 10.1371/journal.pone.0080395 (PMC3832376; doi:10.1371/journal.pone.0080395)
Supplement: Table S1 — PCR primers for this study. (DOCX) [file pone.0080395.s001.docx]

**Table S1.** **PCR primers for this study.**

| Genes | Sequence (5’ to 3’) | Estimated size (bp) |
| --- | --- | --- |
| lacC | Gctactggttgtattggtggaga (F) | 568 |
|  | Attcgctaaacctgaagcgat (R) |  |
| lacE (for type1) | Agcagcgatgcctgctatt (F) | 1335 |
|  | Aagagcattagcaagcatagcg (R) |  |
| lacG (for type1) | CAACAGCAGCCTACCAAGTTG (F) | 1035 |
|  | Cgaagaagcaagtcatacagacc (R) |  |
| lacE (for type2) | Attcgtgatggtttcatcgct (F) | 1195 |
|  | Gtacctgctgcttcttcagca (R) |  |
| lacG (for type2) | Cactgctgaaccagcaagtg (F) | 1108 |
|  | Ttgcactatctgcaatcgca (R) |  |
| sorC | TTCAGACTCATCGACCACTCATT (F) | 702 |
|  | CCAGCATTCCAGTTCCTGTATAA (R) |  |
| sorE | TTGGCATCAGTGGTGACAGA (F) | 1045 |
|  | TTCACTTCACTTAACGCCATTCT (R) |  |
| galK | actgtaactggcgctttcaact (F) | 480 |
|  | caacaactgagtcagcgaatgt (R) |  |
| ptsG | cgttcaactcttgttgctttagtc (F) | 920 |
|  | acaagcatcaacatcagcaatg (R) |  |
| Cas1 | ggatgcttctcggacagaaat (F) | 599 |
|  | Ataggacgaaacggttccataa (R) |  |

*The *lac*E and *lac*G have sequence homology with 64.6% and 66.7% between type I and type II.
