# Supplementary material for: Comparative Genomic Characterization of Three Streptococcus parauberis Strains in Fish Pathogen, as Assessed by Wide-Genome Analyses
Source: PLoS One. 2013 Nov 18;8(11):e80395. doi: 10.1371/journal.pone.0080395 (PMC3832376; doi:10.1371/journal.pone.0080395)
Supplement: Figure S1 — Sugar utilization. Metabolic pathway in three S. parauberis for the conversion of lactose (two Japanese strains) and sorbose (A Korean strain) to glycosysis. (DOCX) [file pone.0080395.s002.docx]

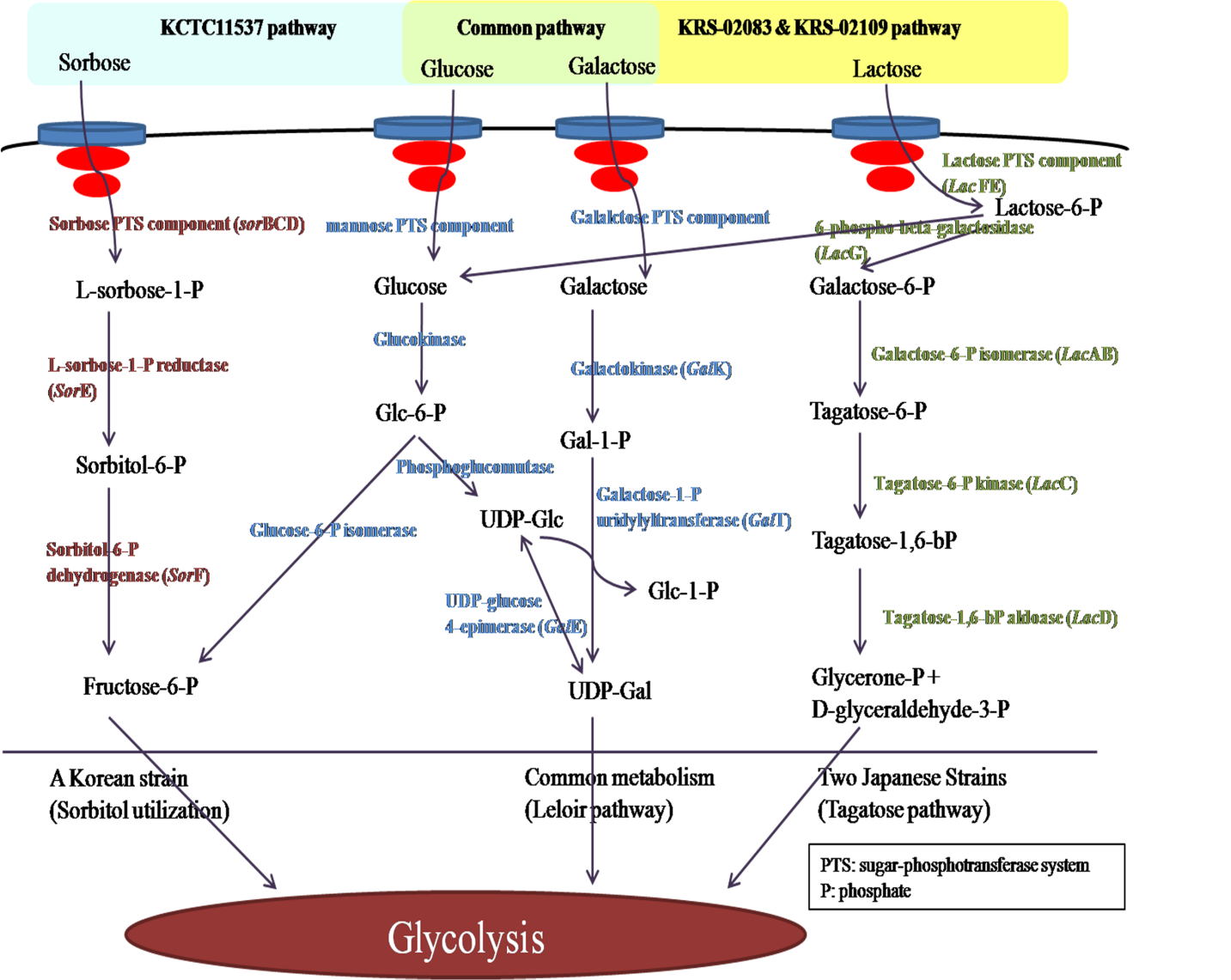


**Figure S1. Sugar utilization.** Metabolic pathway in three *S. parauberis* for the conversion of lactose (two Japanese strains) and sorbose (A Korean strain) to glycosysis.
